# Insulin-like growth factor 1 (IGF1), IGF binding protein 3 (IGFBP3), and breast cancer risk: pooled individual data analysis of 17 prospective studies

**DOI:** 10.1016/S1470-2045(10)70095-4

**Published:** 2010-06

**Authors:** The Endogenous Hormones and Breast Cancer Collaborative Group

## Abstract

**Background:**

Insulin-like growth factor 1 (IGF1) stimulates mitosis and inhibits apoptosis. Some published results have shown an association between circulating IGF1 and breast-cancer risk, but it has been unclear whether this relationship is consistent or whether it is modified by IGF binding protein 3 (IGFBP3), menopausal status, oestrogen receptor status or other factors. The relationship of IGF1 (and IGFBP3) with breast-cancer risk factors is also unclear. The Endogenous Hormones and Breast Cancer Collaborative Group was established to analyse pooled individual data from prospective studies to increase the precision of the estimated associations of endogenous hormones with breast-cancer risk.

**Methods:**

Individual data on prediagnostic IGF1 and IGFBP3 concentrations were obtained from 17 prospective studies in 12 countries. The associations of IGF1 with risk factors for breast cancer in controls were examined by calculating geometric mean concentrations in categories of these factors. The odds ratios (ORs) with 95% CIs of breast cancer associated with increasing IGF1 concentrations were estimated by conditional logistic regression in 4790 cases and 9428 matched controls, with stratification by study, age at baseline, and date of baseline. All statistical tests were two-sided, and a p value of less than 0·05 was considered significant.

**Findings:**

IGF1 concentrations, adjusted for age, were positively associated with height and age at first pregnancy, inversely associated with age at menarche and years since menopause, and were higher in moderately overweight women and moderate alcohol consumers than in other women. The OR for breast cancer for women in the highest versus the lowest fifth of IGF1 concentration was 1·28 (95% CI 1·14–1·44; p<0·0001). This association was not altered by adjusting for IGFBP3, and did not vary significantly by menopausal status at blood collection. The ORs for a difference in IGF1 concentration between the highest and lowest fifth were 1·38 (95% CI 1·14–1·68) for oestrogen-receptor-positive tumours and 0·80 (0·57–1·13) for oestrogen-receptor-negative tumours (p for heterogeneity=0·007).

**Interpretation:**

Circulating IGF1 is positively associated with breast-cancer risk. The association is not substantially modified by IGFBP3, and does not differ markedly by menopausal status, but seems to be confined to oestrogen-receptor-positive tumours.

**Funding:**

Cancer Research UK.

## Introduction

Insulin-like growth factor 1 (IGF1) is a peptide which stimulates mitosis and inhibits apoptosis.^1^, ^2^ Interest in the role of IGF1 in the development of breast cancer began in the 1980s.^3^, ^4^ An early case-control study reported higher plasma concentrations of IGF1 in women with breast cancer than in controls,^5^ and in the first prospective study plasma concentrations of IGF1 were positively associated with breast-cancer risk for premenopausal women, but not for postmenopausal women.^6^ Some, but not all, subsequent prospective studies have supported a positive association between IGF1 and breast-cancer risk, but have been inconsistent as to whether the association differs according to menopausal status.^7^, ^8^, ^9^, ^10^, ^11^, ^12^, ^13^, ^14^, ^15^, ^16^, ^17^, ^18^, ^19^, ^20^, ^21^, ^22^

Around 99% of IGF1 circulates bound to IGF binding proteins, with most bound to IGF binding protein 3 (IGFBP3) in a ternary complex with an acid labile subunit. Less than 1% of IGF1 circulates unbound.^1^ Most prospective studies of IGF1 and breast-cancer risk have also reported on IGFBP3, to explore the hypothesis that women with a high concentration of IGF1 relative to IGFBP3 are at an increased risk of breast cancer.^23^ However, the results of these analyses have been inconsistent. Fewer studies have measured free IGF1, IGF2 or other IGFBPs, such as IGFBP1 and IGFBP2.

Oestrogens are important in the aetiology of breast cancer, and there is laboratory evidence for crosstalk in cells between the signalling pathways for oestrogens and IGF1.^24^ It is therefore important to examine whether the association of IGF1 with breast-cancer risk varies according to the oestrogen-receptor status of the tumour or circulating concentrations of oestradiol.

The Endogenous Hormones and Breast Cancer Collaborative Group was established to do pooled analyses of individual data from prospective studies to increase the precision of the estimated associations of endogenous hormones with breast-cancer risk.^25^ In this study we undertook a collaborative analysis of data from 17 studies to investigate the associations of IGF1 and IGFBP3 with breast-cancer risk. We also examined consistency between studies, associations in subgroups including menopausal status at blood collection and oestrogen receptor status, the effects of adjustment of IGF1 and IGFBP3 for each other and for other risk factors, and the joint associations of IGF1, oestradiol, and testosterone with breast cancer risk in postmenopausal women.

## Methods

### Data collection

Studies were eligible for the collaborative analysis if they had prospectively collected blood samples and data on circulating IGF1, IGFBP3, and breast-cancer risk. Potentially eligible studies were identified through PubMed using the terms “IGF1”, “IGFBP3”, and “breast cancer”, by searching the reference lists of identified studies, and by correspondence with study investigators. 17 eligible studies were identified: CLUE I and CLUE II from the USA;^17^ European Prospective Investigation into Cancer and Nutrition (EPIC), from Europe;^16^ Guernsey study, from the UK;^13^ Janus biobank study, from Norway;^20^ Danish Diet, Cancer, and Health study (KKH), from Denmark;^12^ Kaiser Permanente-Orentreich Foundation Study (KP-OFAS) study, from the USA;^9^ Malmö and Northern Sweden studies, from Sweden;^8^ Melbourne Collaborative Cohort Study (MCCS), from Australia;^19^ Nurses' Health Study, from the USA;^6^, ^15^ Nurses' Health Study II, from the USA;^18^ New York University Women's Health Study (NYU WHS), from the USA;^7^ Study of Hormones and Diet in the Etiology of Breast Tumours (ORDET), from Italy;^10^ Prostate, Lung, Colorectal, and Ovarian Cancer Screening Trial (PLCO), from the USA;^22^ Monitoring Project on Cardiovascular Disease Risk Factors (PPHV) and Prospect-EPIC, from the Netherlands;^11^ Study of Osteoporotic Fractures (SOF), from the USA;^26^ and the Women's Health Initiative, Observational Study (WHI-OS), from the USA.^21^

Table 1 summarises the study designs. Details of the recruitment of participants, informed consent, ethics approvals, and definitions of reproductive variables are in the original publications. Collaborators were asked to provide data on concentrations of IGF1 and IGFBP3, and also on the sex hormones oestradiol and testosterone. Details of the assay methods for IGF1 and IGFBP3 are shown in table 2; 10 studies used serum, six used plasma, and one used both, but for convenience we refer to plasma concentrations throughout this paper. Details of the assay methods for the sex hormones are in the original publications. Collaborators also provided data on reproductive, anthropometric, and other characteristics for each woman in their study. Menopausal status at the time of blood collection was defined on the basis of questions about the number of menstrual periods in the previous year and details of any hysterectomy and ovariectomy; the details varied slightly between studies and are in the original publications. Women were excluded from the analyses if they were perimenopausal or of unknown menopausal status, if they were using hormone-replacement therapy or other exogenous sex hormones at the time of blood collection, or if data were missing for dates of birth, blood collection, or diagnosis (for cases).

**Table 1:** Description of studies

	**Recruitment period**	**Fasting status**	**Storage temperature**	**Matching criteria**		
				Age at blood collection	Date of blood sample	Other matching criteria and comments
CLUE I and CLUE II, USA^17^	1974 and 1989	Non-fasting	−70°C	±1 year	±14 days	Participation in one or both cohorts, menopausal status, ethnic group, freeze and thaw history of serum sample
EPIC, Europe^16^	1992–98	Matched	Mostly −196°C*	±6 months	No (incidence density sampling)	Time of day at blood collection, menopausal status, phase of cycle in premenopausal, subcohort
Guernsey, UK^13^	1977–91	Non-fasting	−20°C	±2 years	±1 year	Menopausal status, phase of cycle in premenopausal
Janus Biobank, Norway^20^	1986–97	Non-fasting	−25°C	All 40–42 years	±6 months	Originally unmatched; matched sets created for this analysis
KKH, Denmark^12^	1993–97	Non-fasting	−150°C	Same half-year	No	Known or probable postmenopausal
KP-OFAS, USA^9^	1964–71	Non-fasting	−23°C until 1980 then −40°C	Age	±1 year	Menopausal status
Malmö/Umeå, Sweden^8^	1985–98	Some matched, some non-fasting	−80 C	±1 year	±1 year	Menopausal status
MCCS, Australia^19^	1990–94	Non-fasting	<−120°C	±24 months	±24 months	Originally a case-cohort study; matched sets created for this analysis
Nurses' Health Study, USA^6^, ^15^	1989–90	Matched, mostly fasting	−130°C	Same year of birth	Same month and year	Time of day, menopausal status at blood collection and diagnosis, fasting status
Nurses' Health Study II, USA^18^	1996–99	Matched, mostly fasting	−130°C	Same year of birth	Same month and year	Time of day, menopausal status at diagnosis, fasting status, luteal day of sample
NYU WHS, USA^7^	1985–91	Non-fasting	−80°C	±3 months	±3 months	Menopausal status, phase of cycle in premenopausal
ORDET, Italy^10^	1987–92	12 hour fast prior to collection. Samples taken 07:30–09:00	−80°C	± 5 years	±89 days	Menopausal status, daylight saving period, recruitment centre
PLCO, USA^22^	1993–2001	Non-fasting	−80°C	±24 months	±24 months	Originally a case-cohort study; matched sets created for this analysis
PPHV, Netherlands^11^	1987–91	Non-fasting	−20°C	±1 year	Same month and year	Place of residence
Prospect-EPIC, Netherlands^11^	1993–97	Non-fasting	−80°C initially then −196°C	±1 year	Same month and year	None
SOF, USA^26^	1986–88	Fat-free overnight and morning diet	−120°C	±24 months	±24 months	Originally a case-cohort study; matched sets created for this analysis
WHI-OS, USA^21^	1993–98	Fasting	−70°C	±24 months	±24 months	Originally a case-cohort study; matched sets created for this analysis

* Stored in liquid nitrogen at −196°C, except in Denmark in nitrogen vapour at −150°C, and in Sweden in electric freezers at −80 °C. EPIC=European Prospective Investigation into Cancer and Nutrition. KKH=Danish Diet, Cancer, and Health study. KP-OFAS=Kaiser Permanente-Orentreich Foundation Study. MCCS=Melbourne Collaborative Cohort Study. NYU WHS=New York University Women's Health Study. ORDET=Study of Hormones and Diet in the Etiology of Breast Tumours. PLCO=Prostate, Lung, Colorectal, and Ovarian Cancer Screening Trial. PPHV=Monitoring Project on Cardiovascular Disease Risk Factors. SOF=Study of Osteoporotic Fractures. WHI-OS=Women's Health Initiative, Observational Study.

**Table 2:** Assay methods

	**Sample**	**IGF1 assay**	**Intra-assay CV**	**Inter-assay CV**	**IGFBP3 assay**	**Intra-assay CV**	**Inter-assay CV**
CLUE I and CLUE II, USA^17^	Phase 1 serum; phase 2 plasma	ELISA (DSL)	Serum 4·0%; plasma 3·2%	Serum 5·9%; plasma 5·9%	ELISA (DSL)	Serum 5·9% Plasma 3·7%	Serum 6·0% Plasma 6·5%
EPIC, Europe^16^	Serum	ELISA (DSL)	6·2%	16·2%	ELISA (DSL)	7·2%	9·7%
Guernsey, UK^13^	Serum	ELISA (DSL)	Overall CV 6·6%	..	RIA (in-house)	Overall CV 3·9%	..
Janus Biobank, Norway^20^	Serum	RIA (in-house)	8%	10%	RIA (in-house)	5%	8%
KKH, Denmark^12^	Serum	TRIFMA (DELFIA)	<5%	<10%	IRMA (DSL)	<5%	<10%
KP-OFAS, USA^9^	Serum	RIA (NID)	6·3%	7·0%	IRMA (DSL)	2·1%	5·3%
Malmö/Umeå, Sweden^8^	Heparin plasma	IRMA (DSL)	13·6%	5·3%	IRMA (DSL)	7·1%	3·1%
MCCS, Australia^19^	Heparin plasma	ELISA (DSL)	9%	7%	ELISA (DSL)	8%	3%
Nurses' Health Study, USA^6^, ^15^	Heparin plasma	ELISA (DSL)	8·7%	15·6%	ELISA (DSL)	9·3%	19·4%
Nurses' Health Study II, USA^18^	Heparin plasma	ELISA (DSL)	6·8%	..	ELISA (DSL)	4·2%	..
NYU WHS, USA^7^	Serum	RIA (in-house)	5·9%	7·9%	RIA (in-house)	6·9%	10·8%
ORDET, Italy^10^	Serum	IRMA (DSL)	4·5%	..	IRMA (DSL)	4·3%	..
PLCO, USA^22^	Serum	ELISA (DSL)	4·4% Overall CV 5·1%	..	ELISA (DSL)	2·8% Overall CV 4·8%	..
PPHV, Netherlands^11^	EDTA plasma	IRMA (DSL)	2·6%	6·4%	IRMA (DSL)	1·1%	4·7%
Prospect-EPIC, Netherlands^11^	Citrate plasma	IRMA (DSL)	2·6%	6·4%	IRMA (DSL)	1·1%	4·7%
SOF, USA^26^	Serum	RIA	2·2%	6·4%	IRMA (DSL)	2·2%	8·0%
WHI-OS, USA^21^	Serum	ELISA (DSL)	<10%	8·2%	ELISA (DSL)	<10%	3·6%

IGF1=insulin-like growth factor 1. IGFBP3=IGF-binding protein 3. EPIC=European Prospective Investigation into Cancer and Nutrition. KKH=Danish Diet, Cancer, and Health study. KP-OFAS=Kaiser Permanente-Orentreich Foundation Study. MCCS=Melbourne Collaborative Cohort Study. NYU WHS=New York University Women's Health Study. ORDET=Study of Hormones and Diet in the Etiology of Breast Tumours. PLCO=Prostate, Lung, Colorectal, and Ovarian Cancer Screening Trial. PPHV=Monitoring Project on Cardiovascular Disease Risk Factors. SOF=Study of Osteoporotic Fractures. WHI-OS=Women's Health Initiative, Observational Study. DELFIA=Immunofluorometric assay. EDTA=ethylene diamine tetraacetic acid. ELISA=Enzyme-Linked Immunosorbant Assay. IRMA=Immunoradiometric assay. RIA=Radioimmunoassay. DSL=Diagnostic Systems Laboratories Inc. NID=Nichols Institute Diagnostics. CV=Coefficient of variation. TRIFMA=Time-resolved immunofluorometric assay. IRMA=Immunoradiometric assay.

### Statistical analysis

Of the 17 studies that contributed data, 11 provided data for women who were premenopausal at blood collection, and 15 provided data for women who were postmenopausal at blood collection. Data from premenopausal women and postmenopausal women in the same cohort were treated as separate sub-cohorts. For the cross-sectional analyses, data were included from all women in the original studies who had not been diagnosed with breast cancer (n=10 022); in the analyses of breast-cancer risk the data were arranged in matched sets, and some potential controls were not matched; therefore, the number of controls with data on IGF1 is less in the risk analyses (n=9428).

**Figure 1:**
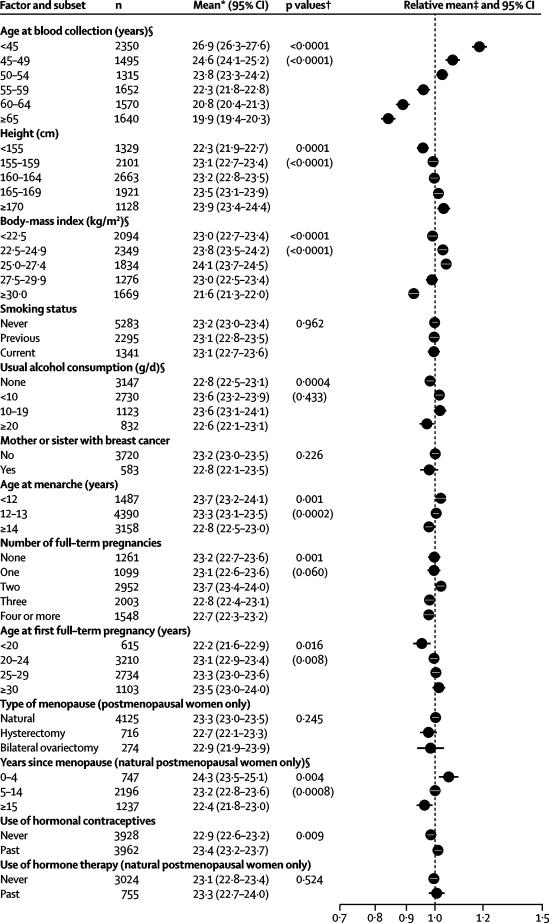
Geometric mean IGF1 concentrations (nmol/L with 95% CI) among controls by selected factors Adjusted for study and age at blood collection, as appropriate. *Means are scaled to the overall geometric mean concentration. †p values for tests of heterogeneity and, where applicable and in parenthesis, linear trend. ‡Values are depicted as a proportion of the overall geometric mean concentration (dotted line). §p<0·05 for test of interaction with study.

Concentrations of IGF1 and IGFBP3 were positively skewed; therefore, log-transformed concentrations were used for all parametric analyses. Correlations between IGF1 and IGFBP3 among premenopausal and postmenopausal controls were calculated using standardised log-transformed concentrations within each study, the standardised values being calculated by subtracting the mean log concentration and dividing by the standard deviation of the log concentration. The associations of IGF1 with risk factors for breast cancer were examined in the controls using linear regression, calculating geometric mean concentrations and 95% CIs according to categories of these factors. Geometric means were adjusted for study and age (age categories as in figure 1), as appropriate. F tests were used to test for heterogeneity in the geometric mean hormone concentrations between the categories of risk factors, and where appropriate to test for trends across the categories, with the ordered categories scored from 1 to the maximum number of categories. The heterogeneity between studies in the associations of IGF1 with risk factors was assessed by adding a study factor interaction term to the model and using the F test to calculate its significance. A similar approach was used to assess heterogeneity according to menopausal status.

11 of the original studies contributing to the collaborative analysis had used matched nested case-control designs, and the remaining six had used unmatched controls or a case-cohort design (Janus,^20^ KKH Denmark,^12^ MCCS,^19^ PLCO,^22^ SOF,^26^ and WHI-OS^21^). Some used density sampling, meaning that an individual participant could appear more than once in a data file; in order to avoid double-counting women in the cross-sectional analyses, we created a pooled dataset in which duplicate observations were deleted, with the case observation retained where a participant appeared as both a case patient and a control. We retained the original matched sets where available, otherwise for the case-cohort studies we created new matched sets in which each case was matched with up to four controls, matching by study, date of blood collection (plus or minus 24 months), age at blood collection (plus or minus 24 months) and, for Janus only,^20^ county of residence.

Conditional logistic regression was used to calculate the odds ratio (OR) for breast cancer in relation to the plasma concentrations of IGF1 and IGFBP3, categorising women in each study according to the quintiles of hormone concentration for the controls in that study. Study-specific cut-points were used because the absolute concentrations of IGF1 and IGFBP3 vary between studies because of laboratory variation; further explanation of this approach is provided in previous publications.^25^, ^27^ To provide a summary measure of risk, we calculated a linear trend by scoring the fifths of the plasma IGF1 or IGFBP3 concentrations as 0, 0·25, 0·5, 0·75, and 1; under the assumption of linearity, a unit change in this trend variable is equivalent to the OR comparing the highest with the lowest fifth of hormone concentration.^27^ Heterogeneity in linear trends among studies was assessed using a χ^2^ test, calculating the χ^2^ statistic as the difference between the sum of the model χ^2^ values for each study and the model χ^2^ value from the all-studies analysis. We also used χ^2^ tests to examine whether there was evidence of heterogeneity in the associations of IGF1 with breast-cancer risk according to subgroups defined by menopausal status at blood collection and other factors.

We examined the effect on the association with breast-cancer risk of adjusting IGF1 and IGFBP3 for each other. We also investigated the associations of IGF1 with breast-cancer risk after adjusting, one factor at a time, for various established reproductive and hormonal risk factors for breast cancer: age at menarche (<12, 12–13, ≥14 years); parity (0, 1, 2, 3, ≥4 full-term pregnancies); age at first full-term pregnancy (<20, 20–24, 25–29, ≥30 years); body mass index (BMI; <22·5, 22·5–24·9, 25·0–27·4, 27·5–29·9, ≥30·0 kg/m^2^); previous use of oral contraceptives (never or ever); and, for postmenopausal women only, type of menopause (natural or surgical); time since menopause (0–4, 5–14, ≥15 years; natural postmenopausal women only); previous use of hormone-replacement therapy (never or ever). For postmenopausal women, we also investigated the associations of IGF1 with breast-cancer risk after adjustment for plasma concentrations of oestradiol and testosterone, and the associations of IGF1 with breast-cancer risk with joint classification according to plasma oestradiol and testosterone concentrations.

All statistical tests were two-sided, and statistical significance was set at the 5% level. All analyses were done using Stata version 9.0.

### Role of the funding source

The funding source had no role in study design, data collection, data analysis, data interpretation, or the writing of the report. The members of the writing team had full access to all data in the study. The corresponding author had the final responsibility for the decision to submit for publication.

## Results

Table 3 shows the characteristics of the cases and controls in each study. There were 4790 cases and 9428 matched controls. Mean age at baseline ranged from 35·5 (SD 7·8) to 47·7 (SD 3·1) for premenopausal women, and from 54·3 (SD 6·1) to 71·8 (SD 4·9) for postmenopausal women. Most women had had a full-term pregnancy, and most postmenopausal women had reported a natural menopause. Across the studies, mean BMI ranged from 23·1 (SD 3·5) to 28·4 (SD 6·3) kg/m^2^, and the median time between blood collection and diagnosis ranged from 1 (IQR 0–3) to 17 (IQR 8–18) years. Geometric mean concentrations of IGF1 ranged from 19·3 (95% CI 18·4–20·3) to 33·9 (32·9–34·9) nmol/L for premenopausal women, and from 14·6 (13·9–15·2) to 28·2 (24·9–31·8) nmol/L for postmenopausal women. Geometric mean concentrations of IGFBP3 ranged from 68·0 (95% CI 65·7–70·4) to 179·4 (175·9–183·0) nmol/L for premenopausal women and from 69·7 (66·8–72·7) to 160·6 (157·9–163·3) nmol/L for postmenopausal women.

**Table 3:** Participant characteristics by study and case-control status

		**Number**	**Age (years)**	**Nulliparous, n (%)**	**Natural menopause, n (%)**	**BMI (kg/m**^2^**)**	Years to diagnosis*	**Geometric mean (95% CI) IGF1, nmol/L**	**Geometric mean (95% CI) IGFBP3, nmol/L**
**Premenopausal at blood collection**									
CLUE I & CLUE II, USA^17^									
	Cases	87	43·1 (6·1)	5 (19%)	..	26·7 (5·9)	11 (8–22)	25·3 (23·3–27·5)	68·0 (65·7–70·4)
	Controls	87	43·0 (6·0)	2 (8%)	..	25·9 (4·4)	..	24·7 (22·7–26·8)	68·7 (66·3–71·1)
EPIC, Europe^16^									
	Cases	409	45·5 (4·8)	66 (17%)	..	24·9 (4·3)	2 (1–4)	33·9 (32·9–34·9)	119·6 (115·6–123·9)
	Controls	793	45·4 (4·8)	110 (15%)	..	25·3 (4·3)	..	33·7 (32·9–34·4)	116·6 (113·7–119·6)
Guernsey, UK^13^									
	Cases	69	40·6 (4·8)	6 (9%)	..	24·7 (3·7)	14 (10–16)	21·5 (19·8–23·4)	159·3 (151·1–168·0)
	Controls	200	40·5 (4·4)	24 (12%)	..	24·2 (3·9)	..	22·1 (21·3–22·9)	168·7 (164·5–173·0)
Janus Biobank, Norway^20^									
	Cases	323	40·5 (0·5)	..	..	..	4 (2–6)	27·0 (26·2–27·8)	176·8 (172·3–181·5)
	Controls	639	40·6 (0·8)	..	..	..	..	26·2 (25·6–26·8)	179·4 (175·9–183·0)
KP-OFAS, USA^9^									
	Cases	89	35·7 (7·9)	18 (21%)	..	23·9 (5·3)	13 (8–18)	31·7 (29·5–34·2)	81·3 (76·5–86·4)
	Controls	89	35·5 (7·8)	19 (22%)	..	23·6 (4·4)	..	30·5 (28·3–32·8)	79·7 (74·9–84·8)
Malmö-Umeå, Sweden^8^									
	Cases	141	47·1 (5·0)	9 (7%)	..	24·5 (4·0)	2 (1–4)	24·1 (22·6–25·7)	126·3 (121·8–130·9)
	Controls	256	47·1 (4·9)	11 (5%)	..	24·8 (4·1)	..	23·6 (22·5–24·7)	124·8 (121·1–128·6)
MCCS, Australia^19^									
	Cases	160	46·8 (4·2)	38 (24%)	..	26·0 (4·9)	4 (2–7)	23·2 (22·0–24·6)	107·6 (104·1–111·2)
	Controls	594	46·1 (4·0)	94 (16%)	..	26·2 (5·1)	..	24·1 (23·4–24·7)	110·9 (109·1–112·7)
Nurses' Health Study, USA^6^, ^15^									
	Cases	194	47·7 (3·1)	13 (7%)	..	24·5 (4·2)	7 (3–8)	27·4 (26·1–28·8)	127·6 (123·1–132·2)
	Controls	262	47·6 (3·1)	13 (5%)	..	25·4 (5·0)	..	27·0 (25·9–28·1)	129·2 (125·4–133·1)
Nurses' Health Study II, USA^18^									
	Cases	231	43·6 (4·0)	53 (23%)	..	24·9 (5·1)	2 (1–4)	31·0 (29·8–32·2)	172·9 (169·7–176·3)
	Controls	454	43·3 (3·8)	84 (19%)	..	25·3 (6·1)	..	30·8 (29·9–31·8)	172·6 (170·1–175·1)
NYU WHS, USA^7^									
	Cases	172	44·4 (4·8)	78 (51%)	..	23·9 (4·0)	5 (3–6)	26·8 (25·5–28·1)	115·6 (111·0–120·5)
	Controls	483	44·2 (4·7)	175 (41%)	..	24·5 (4·5)	..	26·4 (25·7–27·2)	112·7 (110·3–115·2)
ORDET, Italy^10^									
	Cases	62	44·3 (5·0)	8 (13%)	..	24·1 (3·7)	1 (0–3)	20·9 (19·0–23·0)	127·6 (119·2–136·7)
	Controls	239	43·8 (4·5)	25 (11%)	..	24·4 (4·1)	..	19·3 (18·4–20·3)	120·1 (115·0–125·4)
**Postmenopausal at blood collection**									
CLUE I & CLUE II, USA^17^									
	Cases	73	60·6 (5·2)	5 (12%)	38 (86%)	26·5 (5·5)	7 (3–10)	22·0 (19·8–24·4)	72·6 (69·8–75·4)
	Controls	73	60·3 (5·2)	6 (13%)	36 (77%)	25·3 (5·5)	..	20·3 (18·4–22·4)	69·7 (66·8–72·7)
EPIC, Europe^16^									
	Cases	677	60·1 (5·7)	84 (13%)	558 (82%)	27·2 (4·5)	2 (1–4)	28·1 (27·4–28·9)	119·9 (116·4–123·4)
	Controls	1302	60·1 (5·7)	174 (14%)	1074 (82%)	26·8 (4·7)	..	27·2 (26·7–27·7)	115·3 (112·8–117·7)
Guernsey, UK^13^									
	Cases	47	58·9 (5·8)	12 (26%)	43 (91%)	25·6 (3·5)	13 (10–15)	16·8 (15·1–18·7)	159·6 (149·0–170·9)
	Controls	139	59·0 (5·8)	20 (14%)	132 (95%)	25·2 (3·5)	..	17·0 (16·1–17·9)	159·6 (153·6–165·7)
KKH, Denmark^12^									
	Cases	195	57·5 (4·0)	30 (15%)	164 (85%)	26·3 (4·8)	2 (1–3)	17·2 (16·6–17·9)	149·6 (145·7–153·7)
	Controls	195	57·5 (4·0)	29 (15%)	160 (84%)	26·3 (4·5)	..	16·8 (16·2–17·4)	145·0 (141·4–148·7)
KP-OFAS, USA^9^									
	Cases	27	58·6 (5·9)	5 (21%)	27 (100%)	24·3 (2·2)	17 (8–18)	25·0 (22·2–28·2)	77·9 (71·8–84·6)
	Controls	27	58·6 (5·9)	7 (29%)	27 (100%)	23·1 (3·5)	..	28·2 (24·9–31·8)	77·4 (69·2–86·6)
Malmö-Umeå, Sweden^8^									
	Cases	222	60·6 (5·1)	29 (14%)	199 (90%)	26·5 (4·2)	2 (0–3)	18·1 (17·2–19·1)	122·1 (115·6–129·0)
	Controls	401	60·6 (5·1)	32 (9%)	377 (94%)	25·8 (4·4)	..	17·9 (17·0–18·8)	113·7 (107·9–119·9)
MCCS, Australia^19^									
	Cases	257	61·5 (5·2)	36 (14%)	205 (82%)	27·8 (4·7)	4 (2–6)	20·0 (19·1–20·9)	114·3 (111·1–117·5)
	Controls	993	61·3 (5·1)	118 (12%)	760 (79%)	27·5 (5·0)	..	18·6 (18·2–19·1)	109·4 (107·7–111·1)
Nurses' Health Study, USA^6^, ^15^									
	Cases	239	61·5 (4·7)	13 (6%)	164 (73%)	27·0 (5·4)	3 (1–4)	20·3 (19·4–21·3)	128·8 (124·1–133·7)
	Controls	470	61·6 (4·7)	34 (7%)	333 (74%)	26·4 (4·7)	..	20·1 (19·5–20·8)	130·6 (127·2–134·1)
NYU WHS, USA^7^									
	Cases	98	59·1 (3·5)	24 (30%)	78 (80%)	26·4 (4·2)	4 (3–5)	20·7 (19·4–22·2)	109·4 (103·8–115·4)
	Controls	171	59·0 (3·5)	34 (23%)	138 (81%)	25·6 (4·8)	..	20·5 (19·4–21·8)	107·3 (103·1–111·8)
ORDET, Italy^10^									
	Cases	60	58·7 (4·9)	7 (12%)	50 (83%)	26·3 (3·9)	2 (1–3)	15·2 (13·8–16·7)	128·6 (120·9–136·8)
	Controls	220	58·2 (4·9)	28 (13%)	169 (77%)	26·7 (4·2)	..	15·5 (14·7–16·5)	127·9 (123·9–132·0)
PLCO, USA^22^									
	Cases	386	63·8 (5·2)	34 (9%)	295 (77%)	28·1 (5·2)	3 (1–5)	27·4 (26·5–28·4)	160·3 (157·3–163·5)
	Controls	468	63·6 (5·2)	35 (7%)	361 (77%)	27·5 (5·4)	..	26·8 (26·0–27·7)	160·6 (157·9–163·3)
PPHV, Netherlands^11^									
	Cases	77	54·5 (3·3)	..	77 (100%)	26·4 (4·2)	5 (3–8)	23·9 (21·9–26·1)	135·1 (129·7–140·7)
	Controls	167	54·4 (3·8)	..	167 (100%)	26·4 (4·3)	..	22·0 (20·8–23·3)	130·8 (127·6–134·0)
Prospect-EPIC, Netherlands^11^									
	Cases	15	54·3 (6·1)	1 (7%)	15 (100%)	25·4 (4·1)	2 (2–3)	18·5 (15·3–22·3)	109·0 (99·2–119·8)
	Controls	35	54·4 (6·2)	4 (11%)	35 (100%)	26·4 (4·7)	..	18·9 (16·7–21·5)	107·8 (102·7–113·3)
SOF, USA^26^									
	Cases	101	70·8 (4·7)	18 (18%)	87 (86%)	27·7 (5·3)	2 (1–4)	14·8 (14·0–15·7)	139·5 (133·6–145·6)
	Controls	235	71·8 (4·9)	53 (23%)	203 (86%)	26·5 (4·3)	..	14·6 (13·9–15·2)	136·1 (132·0–140·4)
WHI-OS, USA^21^									
	Cases	379	65·8 (7·2)	55 (15%)	268 (71%)	28·4 (6·3)	3 (2–4)	17·3 (16·7–17·9)	144·4 (141·5–147·4)
	Controls	436	64·5 (7·4)	65 (15%)	282 (65%)	27·7 (6·6)	..	17·1 (16·5–17·7)	145·0 (142·5–147·6)

Values are mean (SD) unless otherwise indicated, percentages exclude women with missing values. *Median (inter-quartile range) time between blood collection and diagnosis for cases. Numbers are for women with an IGF1 measurement. EPIC=European Prospective Investigation into Cancer and Nutrition. KKH=Danish Diet, Cancer, and Health study. KP-OFAS=Kaiser Permanente-Orentreich Foundation Study. MCCS=Melbourne Collaborative Cohort Study. NYU WHS=New York University Women's Health Study. ORDET=Study of Hormones and Diet in the Etiology of Breast Tumours. PLCO=Prostate, Lung, Colorectal, and Ovarian Cancer Screening Trial. PPHV=Monitoring Project on Cardiovascular Disease Risk Factors; SOF=Study of Osteoporotic Fractures. WHI-OS=Women's Health Initiative, Observational Study.

Data on IGF1 and IGFBP3 were available for 10 022 and 9889 controls, respectively (these numbers are larger than those for the matched-set analyses because data for unmatched controls were included in the cross-sectional analyses). IGF1 and IGFBP3 were associated with each other, with correlations of 0·38 and 0·50 (data not shown) in premenopausal and postmenopausal women, respectively (both p<0·0001). The associations of IGF1 with selected reproductive and other factors in control women are shown in figure 1 (equivalent analyses for IGFBP3 are in the webappendix p 2). Geometric mean IGF1 was 26% lower for women aged 65 years and above than for women aged less than 45 years; the other results presented in figure 1 are adjusted for age. IGF1 was 7% higher in women who were at least 170 cm tall than in women who were less than 155 cm tall, and was higher in women with a BMI of 25·0–27·4 kg/m^2^ than in thinner or more overweight women. IGF1 was higher in women who drank up to 19 g/d of alcohol than in women who did not drink or who drank at least 20 g/d of alcohol, and was 4% lower for women who had undergone menarche at ages 14 years and over than for women who had undergone menarche before age 12 years. IGF1 varied according to parity, but not in a clear pattern, and was positively associated with age at first full-term pregnancy among parous women. For postmenopausal women, IGF1 was higher for those who had had their menopause most recently. IGF1 was higher for women who had previously used hormonal contraceptives than for those who had not. IGF1 was not significantly associated with smoking, family history of breast cancer, type of menopause, or previous use of hormonal therapy for menopause. The associations of IGF1 with other factors were similar in premenopausal and postmenopausal women (results not shown). Variation in IGFBP3 concentrations by breast-cancer risk factors was less pronounced than that for IGF1 (webappendix p 2).

**Figure 2:**
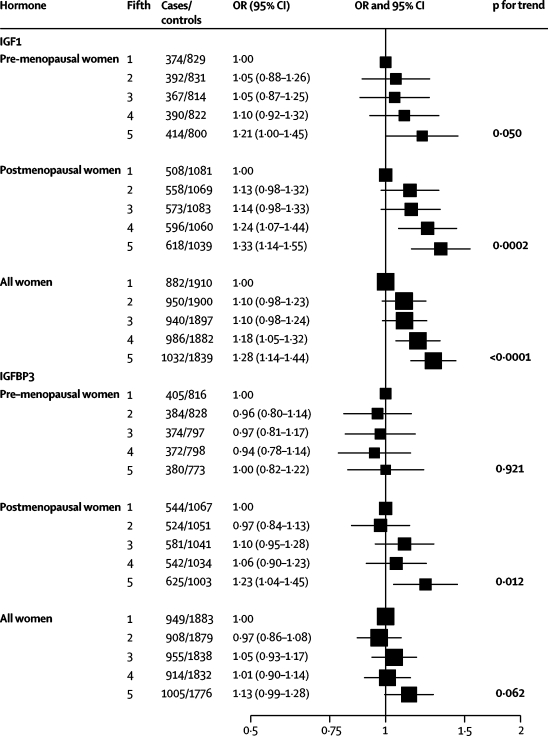
Odds ratios (OR) for breast cancer associated with IGF1 and IGFBP3 among premenopausal women (at blood collection), postmenopausal women (at blood collection), and all women The black squares indicate the ORs and the horizontal lines show the 95% CIs. The area of each square is proportional to the amount of statistical information (inverse of the variance of the logarithm of the OR). Estimates are from conditional logistic regression on case-control sets matched within each study.

**Figure 3:**
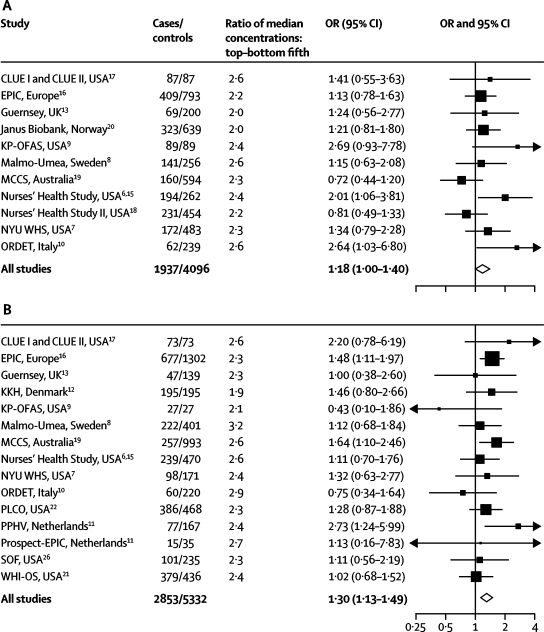
Odds ratios (OR) for breast cancer associated with IGF1 concentrations in women who were premenopausal at blood collection (A), and postmenopausal at blood collection (B) The OR is the estimate of the linear trend for IGF1 obtained by replacing the categorical variables representing the fifths of concentration in controls by a continuous variable scored as 0, 0·25, 0·5, 0·75, and 1. The black squares indicate the ORs and the horizontal lines show the 95% CIs. The area of each square is proportional to the amount of statistical information (inverse of the variance of the logarithm of the OR). The diamonds indicate the OR and 95% CI for all studies combined. Estimates are from conditional logistic regression on case-control sets matched within each study. EPIC=European Prospective Investigation into Cancer and Nutrition. KKH=Danish Diet, Cancer, and Health study. KP-OFAS=Kaiser Permanente-Orentreich Foundation Study. MCCS=Melbourne Collaborative Cohort Study. NYU WHS=New York University Women's Health Study. ORDET=Study of Hormones and Diet in the Etiology of Breast Tumours. PLCO=Prostate, Lung, Colorectal, and Ovarian Cancer Screening Trial. PPHV=Monitoring Project on Cardiovascular Disease Risk Factors. SOF=Study of Osteoporotic Fractures. WHI-OS=Women's Health Initiative, Observational Study.

IGF1 was weakly positively associated with breast-cancer risk for premenopausal women (test for trend, p=0·050) and strongly positively associated with breast-cancer risk for postmenopausal women (test for trend p=0·0002; figure 2); the test for heterogeneity by menopausal status at blood collection was not statistically significant (test for heterogeneity p=0·894). In the individual studies, the ORs for the linear trend for premenopausal women ranged from 0·72 to 2·69, with an overall estimate of 1·18 (95% CI 1·00–1·40), and the median ratio of the IGF1 concentration in the top versus the lowest fifth was 2·3 (figure 3A). The ORs for the linear trend for postmenopausal women ranged from 0·43 to 2·73, with an overall estimate of 1·30 (95% CI 1·13–1·49), and the median ratio of the IGF1 concentration in the top versus the lowest fifth was 2·4 (figure 3B). In the combined analysis of premenopausal and postmenopausal women, those in the highest fifth of IGF1 had an OR of 1·28 (95% CI 1·14–1·44) compared with women in the lowest fifth of IGF1 (test for trend, p<0·0001; figure 2).

IGFBP3 was not associated with breast-cancer risk for premenopausal women (figure 2), but was associated with risk for postmenopausal women (OR in the highest fifth compared with the lowest of 1·23 (95% CI 1·04–1·45, test for trend p=0·012; test for heterogeneity by menopausal status at blood collection p=0·511). In the combined analysis of premenopausal and postmenopausal women, those in the highest fifth of IGFBP3 had an OR of 1·13 (95% CI 0·99–1·28) compared with women in the lowest fifth (test for trend p=0·062).

Measurements of both IGF1 and IGFBP3 in complete matched sets were available for 4727 cases and 9196 controls. Adjustment of the association between IGF1 and breast-cancer risk for IGFBP3 had no significant effect on the OR; the OR for linear trend before adjustment was 1·24 (95% CI 1·11–1·38) and after adjustment was 1·24 (1·10–1·41). By contrast, adjustment of the association between IGFBP3 and breast-cancer risk for IGF1 reduced the OR for linear trend from 1·12 (95% CI 1·00–1·26) to 0·99 (0·87–1·14). Analyses of breast-cancer risk in relation to the molar ratio of IGF1 to IGFBP3 showed a significant positive association, but the magnitude was less than for the analyses of IGF1; ORs in increasing fifths of the ratio were 1·17 (95% CI 1·04–1·32), 1·12 (0·99–1·26), 1·14 (1·01–1·29) and 1·23 (1·08–1·40) (test for trend p=0·009). Further stratified analyses showed that IGFBP3 was not associated with breast-cancer risk within thirds of IGF1 (webappendix p 6).

**Figure 4:**
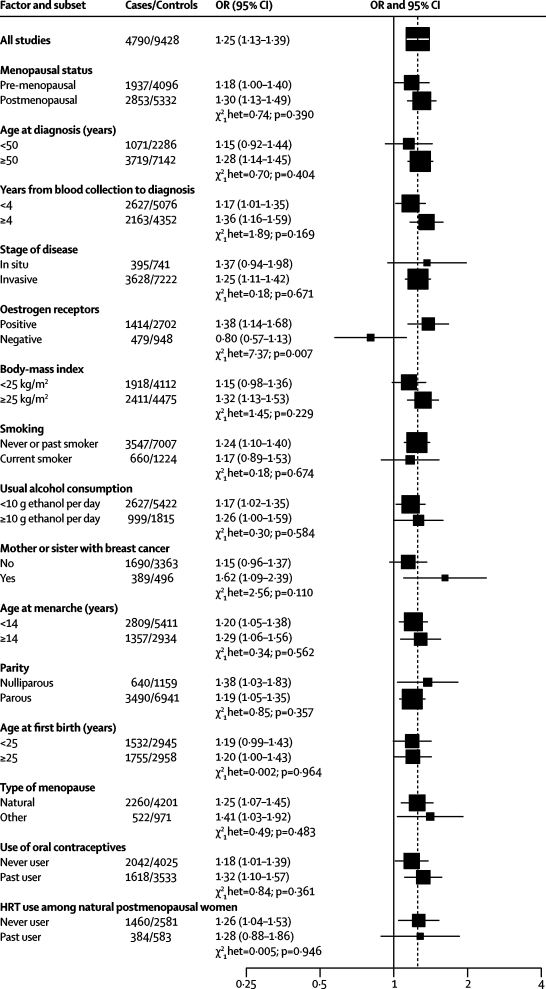
Odds ratios (OR) for breast cancer associated with IGF1 concentration, according to menopausal status at blood collection and other factors The OR is the estimate of the linear trend obtained by replacing the categorical variables representing the fifths of IGF1 concentration in controls by a continuous variable scored as 0, 0·25, 0·5, 0·75 and 1. Black squares indicate the OR and the horizontal lines show the 95% CIs. The area of each square is proportional to the amount of statistical information (inverse of the variance of the logarithm of the OR). The vertical dotted line indicates the OR for all studies. Tests for heterogeneity are for the difference in the association of IGF1 with breast-cancer risk between subgroups. Estimates are from conditional logistic regression on case-control sets matched within each study. HRT=Hormone replacement therapy.

Figure 4 shows the associations with breast cancer of an 80 percentile difference in IGF1 according to subgroups of various factors. The ORs varied according to oestrogen-receptor status; the OR for a linear trend in IGF1 was significant among oestrogen-receptor positive cases (OR 1·38, 95% CI 1·14–1·68), but not for oestrogen-receptor negative tumours (OR 0·80, 0·57–1·13) and the test for heterogeneity was significant (p=0·007). For the other factors there was no significant heterogeneity in the association of IGF1 with breast-cancer risk.

We examined the effect on the association of IGF1 with breast-cancer risk of adjustment, one factor at a time, for height, age at menarche, number of full-term pregnancies, age at first full-term pregnancy, use of hormonal contraceptives, type of menopause (postmenopausal only), time since menopause (postmenopausal only), previous use of hormonal therapy for menopause (postmenopausal only), BMI (premenopausal and postmenopausal analysed separately), plasma oestradiol concentration (postmenopausal only), plasma testosterone concentration (postmenopausal only), time of day of blood collection, and the phase of the menstrual cycle at blood collection (premenopausal only). None of these adjustments altered the OR for IGF1 and breast cancer by more than 2% (data not shown) with the exception of adjustment for testosterone in postmenopausal women, which reduced the OR for an 80 percentile difference in IGF1 from 1·30 (95% CI 1·08–1·55) to 1·24 (1·04–1·49).

The relationship of IGF1 with breast-cancer risk for postmenopausal women was examined together with the associations with oestradiol and testosterone (table 4). In both these joint analyses, the OR increased fairly consistently across thirds of concentrations of both IGF1 and the sex hormone, with no significant interaction.

**Table 4:** Relationships of IGF1 with breast-cancer risk among postmenopausal women, according to plasma concentrations of oestradiol and testosterone

	**Low**		**Medium**		**High**	
	Cases/controls	OR (95% CI)	Cases/controls	OR (95% CI)	Cases/controls	OR (95% CI)
**Thirds of oestradiol (test of interaction: χ^2^_4_=2·31, p=0·679)**						
Low	176/474	1·00 (ref)	205/471	1·20 (0·94–1·53)	194/475	1·21 (0·94–1·55)
Medium	160/396	1·25 (0·96–1·63)	225/415	1·61 (1·26–2·06)	196/424	1·38 (1·07–1·77)
High	212/405	1·70 (1·32–2·19)	240/404	1·89 (1·47–2·43)	267/403	2·08 (1·62–2·67)
**Thirds of testosterone (test of interaction: χ^2^_4_=3·79, p=0·436)**						
Low	125/438	1·00 (ref)	156/424	1·27 (0·96–1·66)	157/378	1·47 (1·11–1·95)
Medium	152/404	1·35 (1·03–1·79)	181/389	1·65 (1·25–2·17)	181/392	1·62 (1·23–2·13)
High	175/345	1·88 (1·41–2·49)	214/385	2·01 (1·54–2·64)	227/426	1·91 (1·47–2·49)

IGFBP3 was not significantly associated with breast-cancer risk in any study of premenopausal women, and was significantly positively associated with risk in three out of 15 studies of postmenopausal women (webappendix pp 3,4). There was significant heterogeneity in the association of IGFBP3 with breast-cancer risk according to oestrogen receptor status; IGFBP3 was non-significantly positively associated with risk for oestrogen-receptor-positive breast cancer and non-significantly inversely associated with risk for oestrogen-receptor-negative breast cancer (test for heterogeneity p=0·039; webappendix p 5).

## Discussion

The results of this collaborative analysis show that plasma concentrations of IGF1 are positively associated with breast-cancer risk. The association is not substantially modified by menopausal status at blood collection or by IGFBP3 concentrations, but seems to be confined to oestrogen-receptor-positive tumours.

The strengths of our study are that the data and plasma samples were all collected prospectively, that it includes almost all the available data from published studies worldwide, and that we were able to adjust for other potential risk factors, including endogenous sex hormones. A potential weakness is that the study designs and methods for measuring IGF1 and IGFBP3 and other risk factors were not standardised. IGF1 and IGFBP3 concentrations varied substantially between studies, and this is likely to reflect differences in assay methods. Our analysis allowed for this by defining study-specific quintiles of IGF1 and IGFBP3 concentrations. This method assumes the true concentrations across the quintiles are similar in all the studies, and if this assumption is not correct then the estimates of ORs might be biased.^27^ However, because heterogeneity between studies in risk estimates was not evident, this assumption does seem reasonable. There was some evidence of heterogeneity between studies in some of the cross-sectional analyses, suggesting that caution should be maintained in the interpretation of these analyses.

Previous studies have examined the associations of IGF1 with other factors. Relevant publications are cited below, but it should be noted that some of these are from the studies contributing to this collaborative analysis.

IGF1 was inversely associated with age, with no obvious additional decline in concentrations around age 50 years, suggesting that menopause itself does not have a marked effect on IGF1. This is consistent with previous observations.^5^, ^28^, ^29^, ^30^, ^31^, ^32^

IGF1 was 7% higher in the tallest women than in the shortest women. No significant association of IGF1 with height was noted in two previous analyses in adults,^29^, ^33^ but these results might be compatible with the small association noted in the current analysis. IGF1 was higher in women with a BMI of 25·0 to 27·4 kg/m^2^ than in thinner or more overweight women, as described previously.^34^ Most circulating IGF1 is produced by the liver, and it is possible that a low BMI is associated with low IGF1 synthesis due to a relatively low supply of nutrients to the liver, whereas obesity is associated with low IGF1 synthesis in the liver due to compromised liver function.^35^

IGF1 was not associated with smoking, consistent with previous observations.^29^, ^30^, ^36^ In relation to alcohol, IGF1 was higher for women who drank a small amount than for those who drank no alcohol or those who drank 20 g or more per day. Other observational studies had similar results.^29^, ^37^, ^38^, ^39^, ^40^ In randomised trials, 15 g/d of alcohol had no effect on IGF1 in postmenopausal women, whereas 30 g/d caused a decrease in IGF1 by 9·5% for premenopausal women and by 4·9% for postmenopausal women.^41^, ^42^

IGF1 did not differ between women with or without a first-degree family history of breast cancer. The inverse association we observed between IGF1 and age at menarche has been noted previously.^43^, ^44^ We observed a non-linear association between IGF1 and parity, with the lowest concentrations for women who had four or more full-term pregnancies; previous studies have not reported any associations between IGF1 and parity.^30^, ^43^, ^44^, ^45^ IGF1 was also positively associated with age at first full-term pregnancy. IGF1 was not associated with type of menopause, but in postmenopausal women was higher in those who had menopause most recently. IGF1 was marginally higher for women who had previously used hormonal contraceptives than for women who had not, but did not vary according to previous use of hormonal therapy for menopause. In future analyses we will examine the relationships of IGF1 with endogenous sex hormones.

The associations of IGF1 with height, age at menarche, age at first full-term pregnancy, and time since menopause are compatible with the possibility that these factors affect breast-cancer risk partly through their relationships with IGF1.

IGF1 concentrations were positively associated with breast-cancer risk, with a highly significant trend and no evidence of heterogeneity between studies. Women in the highest fifth of IGF1 had a 28% higher risk of breast cancer than women in the lowest fifth. This association did not vary significantly according to menopausal status at blood collection or according to the risk factors for breast cancer examined, and was not attenuated by adjustment for other risk factors including IGFBP3, reproductive factors, and, for postmenopausal women, BMI, oestradiol, and testosterone. If the association was due to an effect of preclinical tumours on IGF1 (reverse causality),^46^ then it would be expected to be weaker in those with a greater time interval between blood collection and diagnosis. This was not the case, and the association was highly significant in patients from whom blood had been collected at least 4 years before diagnosis.

A previous meta-analysis based on studies published up to 2006 concluded that the association of IGF1 with breast-cancer risk is limited to premenopausal women,^47^ but our analysis includes four large more recent studies with over 1500 additional patients and shows a clear association of IGF1 with breast-cancer risk in postmenopausal women.

Our analyses were all based on a single hormone measure for each woman. Measurements of hormone concentrations are subject to largely random error associated with assay variation and fluctuations in plasma concentrations within individual women. Five studies have reported the reproducibility of IGF1 over periods of between 1 and 15 years in samples of between 13 and 138 women. The correlations (intra-class or Spearman) between baseline and repeat measures ranged from approximately 0·4 to 0·9 over 1 to 15 years.^10^, ^17^, ^19^, ^48^, ^49^, ^50^ It is therefore likely that the observed association between IGF1 concentrations and breast-cancer risk is an underestimate of the true association, but more reproducibility data are required.

The association of IGF1 with breast-cancer risk was confined to oestrogen-receptor-positive tumours. Further work is needed to examine the potential biological basis for this observation. Laboratory studies have shown that oestrogen increases IGF receptor levels in breast-cancer cells,^51^ whereas in oestrogen-receptor-negative breast-cancer cells the levels of IGF1 receptor are decreased, and IGF1 is non-mitogenic.^52^

IGFBP3 was positively associated with breast-cancer risk, but this association was weak, and was eliminated by adjustment for IGF1, suggesting that the association of IGFBP3 with risk is due to its positive correlation with IGF1. It seems that, at least in the current dataset, the IGFBP3 measures do not add substantial information in assessing the relationship of IGF1 with breast-cancer risk. In addition to its role in transporting IGF1, laboratory studies have shown that IGFBP3 can have direct effects on cell behaviour which can promote apoptosis, but under other circumstances can act against apoptosis.^53^ Data on IGFBP-1 and IGFBP-2 have also been contributed for our collaborative analyses, but currently there are too few data to provide robust analyses. Better understanding of the roles of IGF-binding proteins as potential modulators of the association between IGF1 and breast-cancer risk might come from further data on IGFBP1 and IGFBP2, from measures of intact IGFBP3,^54^ or from measures of bioavailable IGF1.^55^

The OR for IGF1 is smaller than the ORs for both oestrogens and androgens and breast-cancer risk in postmenopausal women, which have been shown in data mostly from the same epidemiological studies; high concentrations of oestradiol and testosterone are associated with around a doubling in breast-cancer risk.^25^, ^56^, ^57^, ^58^ In our analyses, adjustment for oestradiol and testosterone had little effect on the association of IGF1 with breast-cancer risk for postmenopausal women, and there was no evidence of an interaction between IGF1 and oestradiol or testosterone in relation to breast-cancer risk. Nevertheless, a better understanding of the joint effects of hormones on breast-cancer risk is needed.^59^

The association of IGF1 with breast-cancer risk was not altered by adjusting for age at menarche, parity, age at first full-term pregnancy, use of exogenous hormones, and BMI, suggesting that the relationship of IGF1 with breast-cancer risk is not confounded by these other risk factors.

This collaborative analysis has confirmed a positive association between IGF1 and breast cancer risk. It is not known whether this association is causal, but there are plausible biological mechanisms that could explain such an effect.^1^, ^2^ The magnitude of the observed association is modest, but the true association could be substantially larger because of measurement error, and further work is needed to reliably quantify the relationship. If the association is causal then it might have important implications for prevention. Plasma concentrations of IGF1 are influenced by nutritional factors such as energy and protein intake,^60^ and the possibility of lowering breast-cancer risk by reducing IGF1 should be explored.

## Web Extra Material

Supplementary webappendix
