# Supplementary material for: Insulin-like growth factor 1 (IGF1), IGF binding protein 3 (IGFBP3), and breast cancer risk: pooled individual data analysis of 17 prospective studies
Source: Lancet Oncol. 2010 Jun;11(6):530–42. doi: 10.1016/S1470-2045(10)70095-4 (PMC3113287; doi:10.1016/S1470-2045(10)70095-4)
Supplement: Supplementary webappendix [file mmc1.pdf]

THE LANCET Oncology

Supplementary webappendix

This webappendix formed part of the original submission and has been peer reviewed. We post it as supplied by the authors.

Supplement to: The Endogenous Hormones and Breast Cancer Collaborative Group. Insulin-like growth factor 1 (IGF1), IGF binding protein 3 (IGFBP3), and breast cancer risk: pooled individual data analysis of 17 prospective studies. *Lancet Oncol* 2010; published online May 17. DOI:10.1016/S1470-2045(10)70095-4.

Insulin-like growth factor-I (IGF-I), IGF binding protein-3 (IGFBP-3), and breast cancer risk: reanalysis of seventeen prospective studies

Endogenous Hormones and Breast Cancer Collaborative Group

Supplementary Fig. 1.

Geometric mean IGFBP-3 (nmol/L) among controls by selected factors, adjusted for study and, where appropriate, age at blood collection

Factor and subset	n	Mean* (95% CI)	P values[#]
**Age at blood collection[@]**			
under 45	2318	129 (126-131)	0.006
45-49	1477	128 (125-130)	(0.001)
50-54	1301	129 (127-131)	
55-59	1621	125 (123-127)	
60-64	1546	125 (123-127)	
65 and over	1626	122 (120-125)	
**Height (cm)**			
<155	1294	125 (123-127)	0.701
155-159	2072	127 (125-128)	(0.781)
160-164	2628	127 (125-128)	
165-169	1893	126 (125-128)	
170+	1120	126 (124-128)	
**Body mass index (kg/m^2)**			
<22.5	2071	125 (123-126)	0.105
22.5-24.9	2316	126 (124-127)	(0.055)
25.0-27.4	1808	128 (126-130)	
27.5-29.9	1261	127 (125-129)	
30.0+	1631	127 (125-128)	
**Smoking status**			
never	5203	127 (126-128)	0.487
previous	2262	126 (125-128)	
current	1320	125 (123-127)	
**Usual alcohol consumption**			
none	3079	124 (122-125)	<0.0001
<10 g/d	2694	127 (126-129)	(0.0001)
10-19 g/d	1103	129 (127-132)	
20+ g/d	823	129 (126-131)	
**Mother or sister with breast cancer[@]**			
no	3718	126 (125-128)	0.370
yes	582	125 (122-128)	
**Age at menarche**			
under 12	1471	129 (128-131)	<0.0001
12-13	4325	127 (126-128)	(<0.0001)
14 and over	3105	124 (123-125)	
**Number of full-term pregnancies**			
none	1248	125 (123-127)	0.768
one	1091	126 (123-128)	(0.331)
two	2896	127 (125-128)	
three	1977	126 (125-128)	
four or more	1527	127 (125-128)	
**Age at first full-term pregnancy**			
under 20	605	124 (121-127)	0.420
20-24	3160	126 (125-127)	(0.238)
25-29	2691	127 (125-128)	
30 and over	1089	127 (124-129)	
**Type of menopause (postmenopausal women only)[@]**			
natural	4058	127 (126-128)	0.072
hysterectomy	705	124 (122-127)	
bilateral ovariectomy	273	122 (118-127)	
**Years since menopause (natural postmenopausal women only)**			
0-4	733	124 (121-128)	0.464
5-14	2160	127 (125-128)	(0.385)
15+	1222	127 (124-129)	
**Use of hormonal contraceptives**			
never	3861	126 (124-127)	0.163
past	3895	127 (126-128)	
**Use of hormone therapy (natural postmenopausal women only)[@]**			
never	2966	126 (124-127)	0.067
past	746	129 (126-131)	

Relative mean[+] & 95% CI

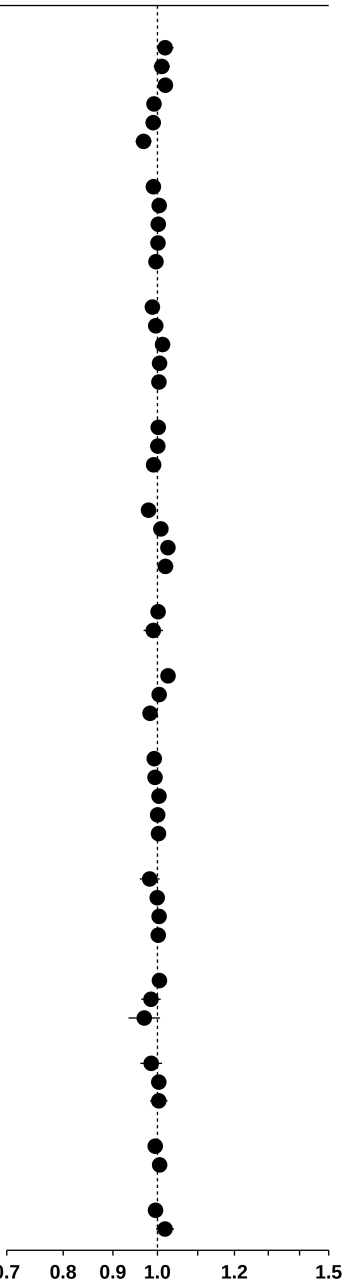

* means are scaled to the overall geometric mean concentration
[#] P values for tests of heterogeneity and, where applicable and in parenthesis, linear trend
[+] values are depicted as a proportion of the overall geometric mean concentration (dotted line)
[@] P<0.05 for test of interaction with study

Webfigure 1. Geometric mean IGFBP-3 concentrations (nmol/l with 95% confidence intervals) among controls by selected factors. Adjusted for study and age at blood collection, as appropriate.

Supplementary Fig. 2a.

Association of breast cancer risk with IGFBP-3 concentration among premenopausal women, by study

Study	Cases/ Controls	Ratio of median concentrations: top-bottom fifth	OR (95% CI)
CLUE I & CLUE II, USA[17]	87/87	1.5	0.80 (0.31-2.03)
EPIC, Europe[16]	392/742	2.5	1.24 (0.64-2.41)
Guernsey, UK[13]	70/203	1.6	0.53 (0.24-1.19)
Janus Biobank, Norway[20]	323/635	1.8	0.88 (0.59-1.33)
KP-OFAS, USA[9]	89/89	2.0	1.33 (0.56-3.15)
Malmo-Umea, Sweden[8]	141/256	1.6	0.96 (0.51-1.78)
MCCS, Australia[19]	154/562	1.6	0.67 (0.40-1.11)
Nurses' Health Study, USA[6,15]	194/262	1.9	1.31 (0.69-2.48)
Nurses' Health Study II, USA[18]	231/454	1.5	1.04 (0.65-1.67)
NYU WHS, USA[7]	172/483	1.8	1.35 (0.79-2.32)
ORDET, Italy[10]	62/239	1.7	1.79 (0.70-4.58)
All studies	1915/4012		0.99 (0.83-1.19)

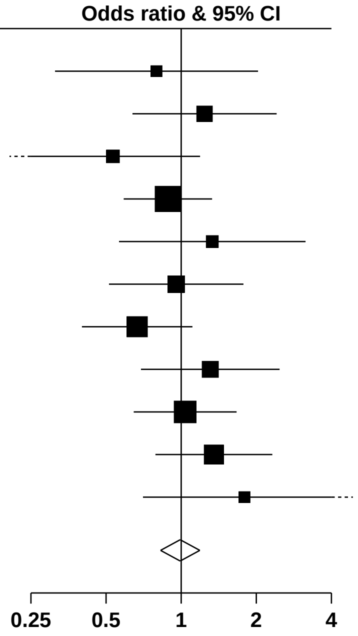

Test of significance (all studies): χ^2_1 = 0.01; P=0.921

Test of heterogeneity between studies: χ^2_{10} = 9.73; P=0.464

The odds ratio is the estimate of the linear trend for IGFBP-3 concentration obtained using a variable that was scored 0, 0.25, 0.5, 0.75, and 1, corresponding to the fifths of concentration.

Webfigure 2a. Odds ratios (ORs) for breast cancer associated with an increase in IGFBP-3 concentration among women who were premenopausal at blood collection. The OR is the estimate of the linear trend for IGFBP-3 obtained by replacing the categorical variables representing the fifths of concentration among controls by a continuous variable scored as 0, 0.25, 0.5, 0.75 and 1. The black squares indicate the ORs and the horizontal lines show the 95% confidence intervals. The area of each square is proportional to the amount of statistical information (inverse of the variance of the logarithm of the OR). The diamond indicates the OR and 95% CI for all studies combined. Estimates are from conditional logistic regression on case-control sets matched within each study.

Supplementary Fig. 2b.
Association of breast cancer risk with IGFBP-3 concentration among postmenopausal women, by study

Study	Cases/ Controls	Ratio of median concentrations: top-bottom fifth	OR (95% CI)
CLUE I & CLUE II, USA[17]	73/73	1.5	1.99 (0.79-4.99)
EPIC, Europe[16]	647/1205	2.6	1.74 (1.13-2.67)
Guernsey, UK[13]	47/139	1.7	1.21 (0.44-3.32)
KKH, Denmark[12]	195/195	1.6	1.78 (0.89-3.55)
KP-OFAS, USA[9]	27/27	2.1	1.17 (0.25-5.54)
Malmo-Umea, Sweden[8]	222/401	2.4	1.10 (0.64-1.89)
MCCS, Australia[19]	252/956	1.8	1.60 (1.07-2.39)
Nurses' Health Study, USA[6,15]	239/470	2.0	0.62 (0.37-1.05)
NYU WHS, USA[7]	98/171	1.9	1.37 (0.63-2.99)
ORDET, Italy[10]	60/220	1.8	0.83 (0.36-1.89)
PLCO, USA[22]	386/468	1.6	1.12 (0.76-1.65)
PPHV, Netherlands[11]	77/167	1.5	2.48 (1.06-5.84)
Prospect-EPIC, Netherlands[11]	15/35	1.5	0.94 (0.16-5.33)
SOF, USA[26]	101/235	1.7	1.17 (0.59-2.32)
WHI-OS, USA[21]	377/434	1.6	0.85 (0.57-1.26)
All studies	2816/5196		1.21 (1.04-1.41)

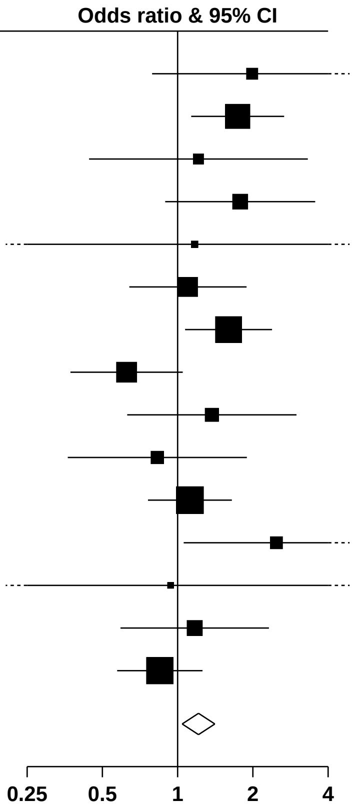

Test of significance (all studies): χ^2_1 = 6.30; P=0.012

Test of heterogeneity between studies: χ^2_{14} = 20.59; P=0.113

The odds ratio is the estimate of the linear trend for IGFBP-3 concentration obtained using a variable that was scored 0, 0.25, 0.5, 0.75, and 1, corresponding to the fifths of concentration.

Webfigure 2b. Odds ratios (ORs) for breast cancer associated with an increase in IGFBP-3 concentration among women who were postmenopausal at blood collection. The OR is the estimate of the linear trend for IGFBP-3 obtained by replacing the categorical variables representing the fifths of concentration among controls by a continuous variable scored as 0, 0.25, 0.5, 0.75 and 1. The black squares indicate the ORs and the horizontal lines show the 95% confidence intervals. The area of each square is proportional to the amount of statistical information (inverse of the variance of the logarithm of the OR). The diamond indicates the OR and 95% CI for all studies combined. Estimates are from conditional logistic regression on case-control sets matched within each study.

Supplementary Fig. 3.

Odds ratios associated with IGFBP-3 according to menopausal status and other factors

Factor and subset	Cases/Controls	OR (95% CI)
**All studies**	**4731/9208**	**1.12 (0.99-1.25)**
**Menopausal status**		
**premenopausal**	**1915/4012**	**0.99 (0.83-1.19)**
**postmenopausal**	**2816/5196**	**1.21 (1.04-1.41)**
		χ^2_1 het = 2.82; P=0.093
**Age at diagnosis**		
**<50**	**1055/2232**	**1.01 (0.79-1.28)**
**≥50**	**3676/6976**	**1.15 (1.01-1.31)**
		χ^2_1 het = 0.91; P=0.339
**Years from blood collection to diagnosis**		
**<4**	**2599/4982**	**1.11 (0.94-1.30)**
**≥4**	**2132/4226**	**1.13 (0.95-1.33)**
		χ^2_1 het = 0.03; P=0.870
**Stage of disease**		
**in situ**	**393/736**	**1.62 (1.07-2.46)**
**invasive**	**3582/7036**	**1.06 (0.93-1.21)**
		χ^2_1 het = 3.67; P=0.055
**Oestrogen receptors**		
**positive**	**1405/2654**	**1.15 (0.95-1.40)**
**negative**	**477/938**	**0.77 (0.55-1.07)**
		χ^2_1 het = 4.27; P=0.039
**Body mass index**		
**<25 kg/m^2**	**1902/4032**	**1.08 (0.91-1.28)**
**≥25 kg/m^2**	**2368/4339**	**1.15 (0.98-1.35)**
		χ^2_1 het = 0.36; P=0.547
**Smoking**		
**never or past smoker**	**3499/6819**	**1.13 (0.99-1.29)**
**current smoker**	**650/1198**	**1.20 (0.90-1.59)**
		χ^2_1 het = 0.16; P=0.689
**Usual alcohol consumption**		
**<10 g ethanol per day**	**2578/5248**	**1.09 (0.93-1.27)**
**≥10 g ethanol per day**	**988/1770**	**1.13 (0.89-1.45)**
		χ^2_1 het = 0.09; P=0.770
**Mother or sister with breast cancer**		
**no**	**1689/3365**	**1.03 (0.86-1.23)**
**yes**	**389/495**	**1.35 (0.92-1.99)**
		χ^2_1 het = 1.68; P=0.195
**Age at menarche**		
**<14 years**	**2775/5286**	**1.06 (0.91-1.22)**
**≥14 years**	**1330/2846**	**1.27 (1.04-1.55)**
		χ^2_1 het = 2.46; P=0.117
**Parity**		
**nulliparous**	**633/1135**	**1.14 (0.85-1.52)**
**parous**	**3444/6768**	**1.12 (0.98-1.28)**
		χ^2_1 het = 0.01; P=0.906
**Age at first birth**		
**<25 years**	**1513/2866**	**1.06 (0.88-1.29)**
**≥25 years**	**1723/2887**	**1.21 (1.00-1.46)**
		χ^2_1 het = 1.07; P=0.301
**Type of menopause**		
**natural**	**2229/4089**	**1.17 (0.99-1.39)**
**other**	**516/952**	**1.34 (0.98-1.85)**
		χ^2_1 het = 0.58; P=0.447
**OC use**		
**never user**	**2008/3913**	**1.11 (0.93-1.31)**
**past user**	**1594/3430**	**1.25 (1.03-1.52)**
		χ^2_1 het = 0.99; P=0.319
**HRT use among natural postmenopausal women**		
**never user**	**1434/2498**	**1.16 (0.94-1.44)**
**past user**	**379/575**	**1.18 (0.80-1.74)**
		χ^2_1 het = 0.00; P=0.946

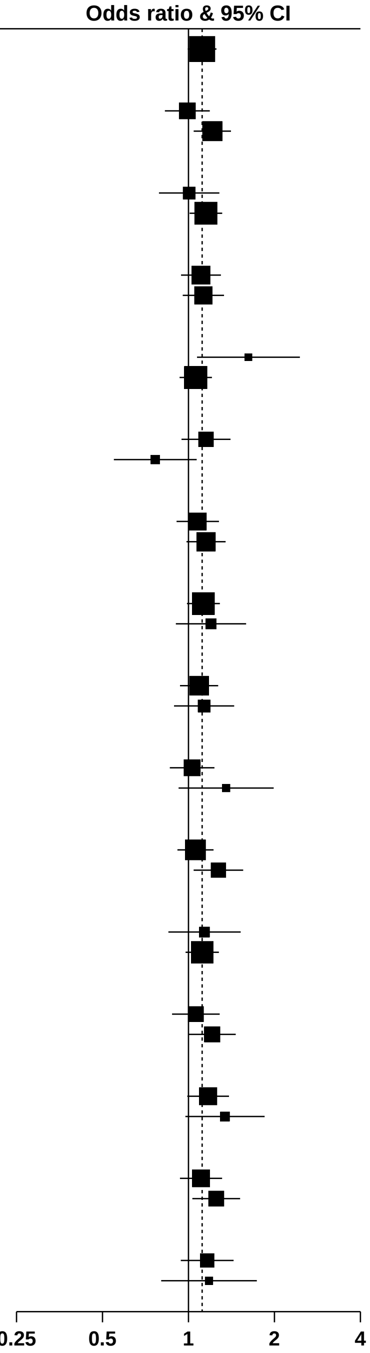

Webfigure 3. Odds ratios (ORs) for breast cancer associated with IGFBP-3 concentration, according to menopausal status at blood collection and other factors. The OR is the estimate of the linear trend obtained by replacing the categorical variables representing the fifths of IGFBP-3 concentration among controls by a continuous variable scored as 0, 0.25, 0.5, 0.75 and 1. Black squares indicate the OR and the horizontal lines show the 95% confidence intervals. The area of each square is proportional to the amount of statistical information (inverse of the variance of the logarithm of the OR). The vertical dotted line indicates the OR for all studies. Tests for heterogeneity are for the difference in the association of IGFBP-3 with breast cancer risk between subgroups. Estimates are from conditional logistic regression on case-control sets matched within each study.

Webtable 1: Relationships of IGF-I with breast cancer risk among all women, according to plasma concentrations of IGFBP-3

Thirds of IGFBP-3	Thirds of IGF-I					
	Low		Medium		High	
	**Cases/controls**	**RR (95% CI)**	**Cases/controls**	**RR (95% CI)**	**Cases/controls**	**RR (95% CI)**
**Low**	825/1730	1.00 (ref)	518/988	1.12 (0.97-1.28)	223/427	1.15 (0.95-1.40)
**Medium**	408/847	0.99 (0.86-1.15)	592/1194	1.06 (0.93-1.21)	572/1018	1.22 (1.06-1.40)
**High**	239/505	0.91 (0.75-1.11)	464/896	1.05 (0.90-1.22)	886/1591	1.18 (1.04-1.34)
	Test of interaction: χ^2_4=1.15, P=0.887					
